# IUC26590-87 Outcomes of Novel Systemic Therapies in Advanced Upper Tract Urothelial Carcinoma: A Systematic Review and Meta-Analysis

**DOI:** 10.1093/oncolo/oyag286.005

**Published:** 2026-08-21

**Authors:** A D'arienzo, D Incognito, S Facchini, C Barraco, A Nicastro, M Berretta, G Facchini

**Affiliations:** Oncology Unit, S. Maria Delle Grazie Hospital, Pozzuoli, Naples - Italy; PhD Student in Translational Molecular Medicine and Surgery, Department of Clinical and Experimental Medicine, University of Messina, 98122 Messina - Italy; Experimental Clinical Abdominal Oncology Unit, Istituto Nazionale Tumori- IRCCS-Fondazione G. Pascale, Naples - Italy; Oncology Unit, S. Maria Delle Grazie Hospital, Pozzuoli, Naples - Italy; Oncology Unit, S. Maria Delle Grazie Hospital, Pozzuoli, Naples - Italy; Department of Clinical and Experimental Medicine, University of Messina - Italy; Oncology Unit, S. Maria Delle Grazie Hospital, Pozzuoli, Naples - Italy

## Abstract

**Background:**

Advanced upper tract urothelial carcinoma (UTUC) differs clinically and biologically from bladder carcinoma. Treatment recommendations are largely extrapolated from trials enrolling predominantly lower-tract disease. We performed a systematic review and meta-analysis to assess whether contemporary systemic therapies improve survival in locally advanced or metastatic UTUC.

**Methods:**

We searched PubMed, EMBASE, and major oncology meeting abstracts from Jan 2014 to March 2026. Randomized trials comparing immune checkpoint inhibitors, antibody-drug conjugates, targeted agents or antiangiogenic therapies with chemotherapy or best supportive care were eligible. Hazard ratios (HRs) for overall survival (OS) and progression-free survival (PFS) in UTUC subgroups were pooled using random-effects inverse-variance models.

**Results:**

A total of 45 randomized trials were eligible, but 30 (67%) did not report UTUC-specific survival outcomes. Fifteen trials, including 10,824 patients overall and 2,897 with UTUC, were quantitatively synthesized. UTUC representation ranged from 11.2% to 37.7% in non-biomarker-selected studies. No dedicated trial was restricted to advanced UTUC, underscoring a major evidence gap. In first-line treatment, novel combinations significantly improved OS in UTUC (pooled HR 0.73, 95% CI 0.62–0.85; I² = 8.7%) and PFS (HR 0.62, 95% CI 0.46–0.84; I² = 71.3%) versus platinum-based chemotherapy. Antibody-drug conjugate plus immunotherapy combinations produced the largest effects. Avelumab maintenance showed a numerical but non-significant benefit in UTUC (OS HR 0.90, 95% CI 0.59–1.39; PFS HR 0.85, 95% CI 0.60–1.21). In later lines, novel therapies did not significantly improve OS over chemotherapy (HR 0.91, 95% CI 0.78–1.06). The most pronounced OS benefit was observed with erdafitinib in FGFR2/3-altered, ICI-pretreated UTUC (THOR cohort 1; HR 0.34, 95% CI 0.18–0.64).

**Conclusions:**

Patients with advanced UTUC derive a first-line survival benefit from modern combination therapies comparable to that observed in unselected urothelial carcinoma populations. ADC–immunotherapy combinations appear to be the best treatment option. In later treatment lines, a biomarker-driven strategy is essential to identify patients most likely to benefit from targeted therapies.

**IUC26590-87 Figure 1:**
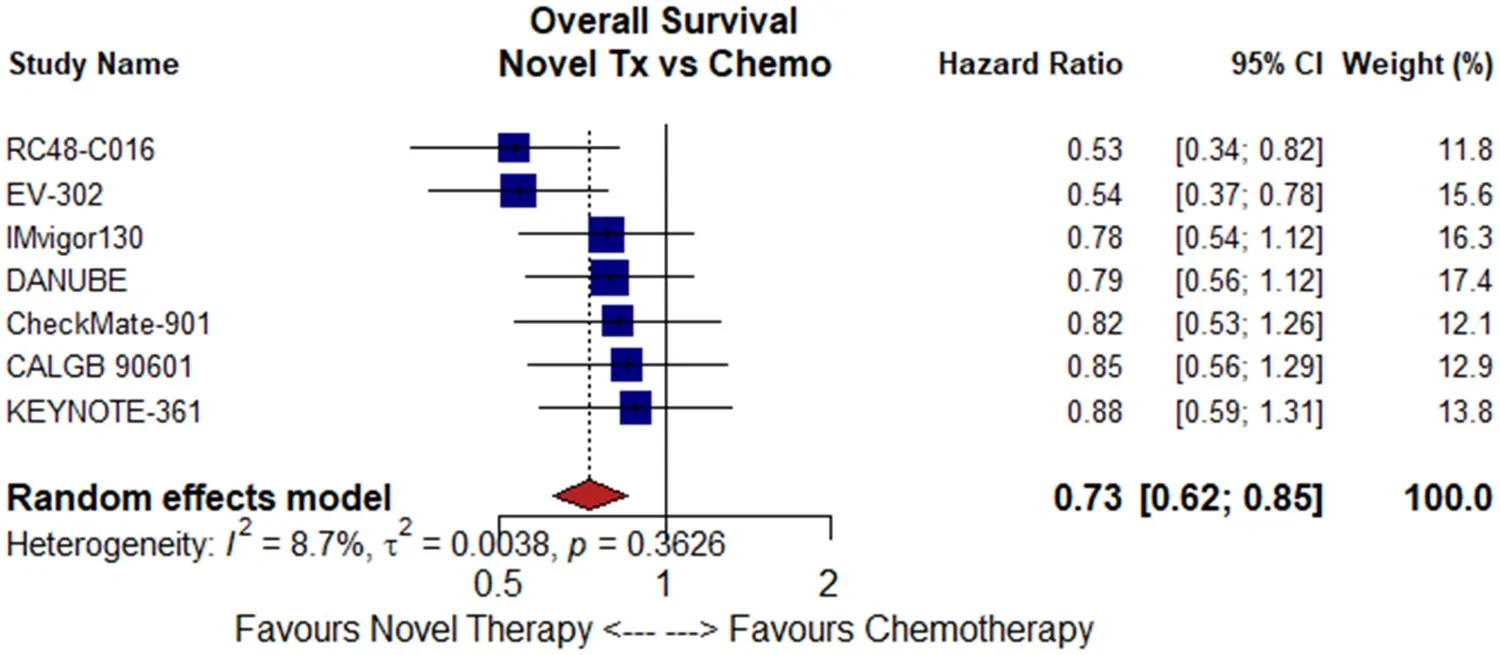
Pooled OS HR for treatment combination in first-line UTUC subgroup

